# Wave-momentum shaping for moving objects in heterogeneous and dynamic media

**DOI:** 10.1038/s41567-024-02538-5

**Published:** 2024-06-21

**Authors:** Bakhtiyar Orazbayev, Matthieu Malléjac, Nicolas Bachelard, Stefan Rotter, Romain Fleury

**Affiliations:** 1 Physics Department, School of Sciences and Humanities, Nazarbayev University, Astana, Kazakhstan; 2 Laboratory of Wave Engineering, School of Engineering, EPFL, Lausanne, Switzerland; 3 Université de Bordeaux, CNRS, LOMA, UMR5798, Talence, France; 4 Institute of Theoretical Physics, Vienna University of Technology (TU Wien), Vienna, Austria

**Keywords:** Acoustics, Electronics, photonics and device physics

## Abstract

Light and sound waves can move objects through the transfer of linear or angular momentum, which has led to the development of optical and acoustic tweezers, with applications ranging from biomedical engineering to quantum optics. Although impressive manipulation results have been achieved, the stringent requirement for a highly controlled, low-reverberant and static environment still hinders the applicability of these techniques in many scenarios. Here we overcome this challenge and demonstrate the manipulation of objects in disordered and dynamic media by optimally tailoring the momentum of sound waves iteratively in the far field. The method does not require information about the object’s physical properties or the spatial structure of the surrounding medium but relies only on a real-time scattering matrix measurement and a positional guide-star. Our experiment demonstrates the possibility of optimally moving and rotating objects to extend the reach of wave-based object manipulation to complex and dynamic scattering media. We envision new opportunities for biomedical applications, sensing and manufacturing.

## Abstract (web summary)

Although manipulation of objects using light and sound waves is an established technique, it has so far been confined to static environments. Iterative tailoring of acoustic far fields now allows control of objects in disordered and dynamic media.

## Main

Ever since the emergence of optical tweezers^1,2^, the non-contact manipulation of objects using electromagnetic^3,4^ and acoustic waves^5–7^ has become a central paradigm in quite diverse fields ranging from optomechanics to bioacoustics. Sound waves, in particular, offer distinct advantages, as they are biocompatible and harmless and their short wavelengths can penetrate a wide range of heterogeneous, opaque and absorbing media. Another key feature of acoustics is its wide frequency range, spanning from hertz to gigahertz, which facilitates the manipulation of particles varying in size from a few centimetres to a few micrometres. In this way, not only Mie^8–10^ and Rayleigh particles can be addressed, but also complex objects including individual biological cells^11–13^.

Although various strategies have already been developed to collectively or selectively manipulate objects and particles, these techniques always rely on controlled and static environments. Collective dynamic positioning of particles trapped in the potential wells of a pressure field has been achieved in one^14^, two^15,16^ and three^17–20^ dimensions. Typically, by generating appropriate standing waves^21^, particles or objects are trapped in the vicinity of the pressure nodes or antinodes, depending on their contrast ratio with the surrounding fluid^13^. More advanced strategies have also been developed to address the selectivity problem of standing-wave-based trapping involving acoustic vortices^22^ or the use of additional systems such as lenses^23^, metasurfaces^24^ or holograms^18,25–28^. Considerable attention has also been paid to the development of on-chip acoustofluidic and acoustophoretic devices^12,29–31^ and wave-controlled microrobots^32–38^ for lab-on-a-chip and biomedical applications. However, the requirements for a precisely controlled static environment and proximity to the target substantially restrict the applicability of these various techniques in many real-world scenarios. Practical cases involve disordered or dynamic environments where manipulation must occur at a considerable distance from the object that needs to be manipulated.

Here, we propose and experimentally demonstrate a wave-momentum shaping approach that requires only far-field information and allows us to move and rotate objects even in disordered or dynamic environments. Instead of relying on potential wells to trap the object, we continuously find and send the optimal mode mixture that transfers an optimal amount of momentum to the object. This mode mixture is updated during the motion as the scattering changes. The method is experimentally demonstrated in a macroscopic two-dimensional acoustic cavity containing a movable object and a collection of scatterers. Far-field scattering matrix measurements allow us to determine the optimal wavefronts for shifting or rotating the object at each moment in time. Remarkably, the method neither requires knowledge or modelling of acoustic forces nor any prior information on the physical properties of the object or disorder. Only a guide-star measurement of the object’s position or rotation angle is needed, which is here provided by a camera. The remarkable robustness of the method is emphasized by implementing it in a dynamic scenario, where the scatterers composing the environment move randomly. The method may be transposed to other platforms and scales, such as ultrasound or light for the motion of microscopic bodies.

## Principles of wave-momentum shaping

The idea of wave-momentum shaping was inspired by recent developments in adaptive optics and disordered photonics, for which wavefront shaping techniques have been significantly advanced to focus light in disordered media or to compensate for aberrations and multiple-scattering for various purposes^39–43^. In the most straightforward implementation, a feedback mechanism allows a quantity of interest, such as the optical power focused at a given point, to be iteratively optimized by tuning the incident wavefronts^39^. On the other hand, more advanced approaches, such as Wigner–Smith operators derived from a system’s scattering matrix, can focus light in disorder optimally^44^, exert a maximal electromagnetic force or torque on static objects^45,46^, or potentially even cool an ensemble of levitated particles^47,48^. Wave-momentum shaping applies these ideas to the manipulation of moving objects. It combines the optimal character of Wigner–Smith approaches with iterative guide-star techniques, necessitated by the dependence of the *S* matrix on the object position, which influences the complex scattering process and constantly modifies the field speckle.

**Fig. 1:**
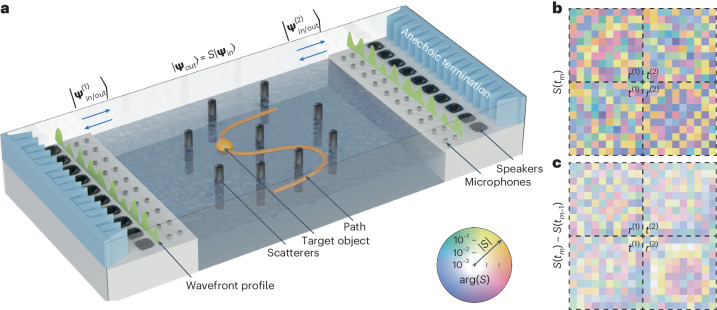
Moving an object in a complex scattering medium by acoustic-wave-momentum shaping. **a**, We consider a parallel-plate acoustic waveguide supporting ten modes at the working frequency. It contains cylindrical rigid scatterers (in black). The bottom surface of this waveguide is formed by the water in this container, so that the spherical object (orange ball) can float and move freely. Wave-momentum shaping consists of finding and sending, at each time *t*_*m*_, the optimal mode mixture to push the ball along an arbitrarily chosen path (orange line). We achieve this by real-time far-field measurements, which allow us to track the evolution of the scattering matrix *S* as the object moves. We deduce the wavefronts to be injected by the external speaker arrays such that they optimally deliver the target momentum. **b**, Example of a scattering matrix measured at a given time *t*_*m*_ in our experiment. **c**, Difference between the scattering matrix at *t*_*m*_ and the one measured at *t*_*m*−1_, showing the influence of the translation of a small object on scattering. We use the information collected at three consecutive time steps to derive the mode mixture that optimally pushes the ball in the desired direction. The static scatterers are later replaced with dynamic ones. Source data

Consider the experimental set-up illustrated in Fig. 1a. It consists of an acoustic multimode waveguide supporting ten modes (*N* = 10) at the operational frequency *f*_0_ = 1,590 Hz (audible sound) corresponding to an acoustic wavelength of λ_0_ ≈ 0.22 m. In the central part, we introduced a movable object (ping-pong ball) with a radius of 20 mm (~0.1*λ*_0_), which floats on the surface of a water tank. Within this tank, several static cylindrical scatterers (depicted as black cylinders) protrude above the water level, thus creating a complex scattering landscape. Two arrays of ten speakers are placed on both sides, labelled 1 and 2, which allow us to control the incident acoustic mode mixtures $\left\vert {{{{\boldsymbol{\Psi }}}}}_\mathrm{in}^{(1,2)}\right\rangle$. These incident waves are linearly scattered into outgoing mode mixtures $\left\vert {{{{\boldsymbol{\Psi }}}}}_\mathrm{out}^{(1,2)}\right\rangle$, which can be measured using microphones placed in the waveguide’s asymptotic regions (Methods and Supplementary Information Sections 1 and 2). From such measurements, it is possible to deduce the scattering matrix *S*(*t*), which evolves with time because, as the target object moves, it modifies the scattering in the central region. This dynamic scattering matrix obeys the relation $\left\vert {{{{\boldsymbol{\Psi}}}}}_\mathrm{out}(t)\right\rangle ={{S}}(t)\left\vert {{{{\boldsymbol{\Psi}}}}}_\mathrm{in}(t)\right\rangle$, where we gathered the states related to both sides into single column vectors.

Figure 1b shows an example of the measured scattering matrix, of dimension 2*N* × 2*N*, which is composed of four *N* × *N* sub-blocks, the reflection and transmission matrices *r*^(1),(2)^ and *t*^(1),(2)^, describing how each of the ten modes scatter on each side. The figure encodes the amplitude of the matrix coefficients in the transparency of the squares and the phase in their colour. Clearly, mode mixing occurs due to the complex scattering. Quite intuitively, to make object manipulation possible, such a scattering matrix must depend on the position of the movable object. This dependence is evidenced by repeating the scattering matrix measurement after slightly moving the object (by a distance equal to a quarter of its radius, *l* = 5 mm). The difference is plotted in Fig. 1c. We observe that although the changes are small in magnitude, consistent with the fact that only one scatterer is moved, some information about the object’s motion seems to be embedded in these changes.

The *S* matrix’s dependence on the object’s position is relevant in the context of its dynamic manipulation. If we denote by *α* either the *x* or *y* coordinate of the movable object, the momentum transferred to it upon scattering Δ*p*_*α*_ can be calculated from the expectation values of the operator *C*_*α*_ = −i∂/∂*α* for the superposition states $\left\vert {{{{\boldsymbol{\Psi }}}}}_\mathrm{in,out}\right\rangle$ (ref. ^45^). The momentum transferred to the particle upon scattering is the difference between the momentum of the outgoing and incident mode mixtures in the vicinity of the particle. Assuming unitary scattering (${{{{{S}}}}}^{{\dagger} }{{{{S}}}}={\mathbb{1}}$), one can demonstrate the following link between this momentum transfer and the variation of *S* with *α* (Methods):

1 $$\Delta {p}_{\alpha}=\left\langle {{{{\boldsymbol{\Psi}}}}}_{{{\mathrm{in}}}}\middle| \left(-{{{\rm{i}}}}{{{S}}}^{-1}\frac{\mathrm{d}S}{\mathrm{d}\alpha}\right)\middle| {{{{\boldsymbol{\Psi}}}}}_{{{\mathrm{in}}}}\right\rangle ,$$

where the Hermitian operator

2 $${{{Q}}}_{\alpha}=-{{{\rm{i}}}}{{{S}}}^{-1}\frac{\mathrm{d}S}{\mathrm{d}\alpha},$$

is known as a generalized Wigner–Smith (GWS) operator^44^. Equation (1) means that the momentum imparted locally onto the moving object upon scattering is related to the expectation value of *Q*_*α*_ for the specific input state $\left\vert {{{{\boldsymbol{\Psi }}}}}_\mathrm{in}\right\rangle$ in the far field. A direct consequence of equation (1) is that if the input state $\left\vert {{{{\boldsymbol{\Psi }}}}}_\mathrm{in}\right\rangle$ is chosen to be an eigenvector of *Q*_*α*_, the momentum kick on the object will be proportional to its eigenvalue. Therefore, choosing the eigenstate with the highest eigenvalue as the input mode mixture will optimize the transfer of momentum to the object in the direction *α*. This is the basic physical principle behind wave-momentum shaping.

## Linear-momentum transfer

We first apply wave-momentum shaping to the transfer of linear momentum and experimentally demonstrate complete control over the trajectory of a moving object in a complex scattering medium, which is static for now. We start from the set-up of Fig. 1 and apply an iterative motion algorithm that works as follows: (1) Initially, the object is at rest. We send three random wave fields **Ψ**_*m*−3_,**Ψ**_*m*−2_ and **Ψ**_*m*−1_ to move it slightly but randomly and measure the *S*_*m*−3_, *S*_*m*−2_ and *S*_*m*−1_ matrices at three different nearby points, whose positions (*x*_*m*−3_, *y*_*m*−3_),(*x*_*m*−2_, *y*_*m*−2_) and (*x*_*m*−1_, *y*_*m*−1_) are measured by a camera. (2) From these measurements, we estimate the components d*S*/d*α* of the gradient of *S* with respect to the coordinates *α* = *x*, *y*, using discrete derivative approximations, equations (3) and (4). (3) We compose *Q*_*α*_ from equation (2) and diagonalize it to obtain mode mixtures and momentum expectations (eigenvalues) in *α* = *x*_*m*_, *y*_*m*_. (4) We send a superposition of eigenvectors of *Q*_*x*_ and *Q*_*y*_ in proportion to move the object in a desired direction and measure *S* again once the object has moved and stabilized. (5) The process is iteratively repeated based on the last three measured *S* matrices until the object arrives at the desired destination. The method does not require calibration or access to the interior of the medium.

**Fig. 2:**
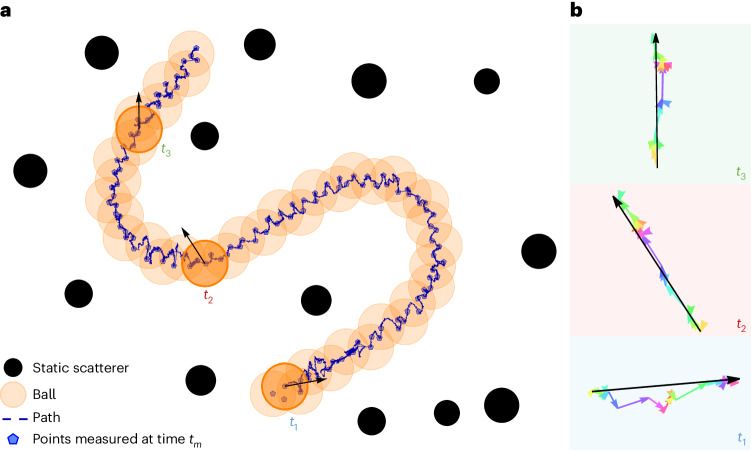
Experimental demonstration of guiding an object through acoustic-wave-momentum shaping in a static scattering medium. **a**, A set of points, in blue, are chosen to define an overall S-shaped path to be followed by the moving ball, whose successive positions, as captured by a camera, are shown by orange discs. The ball successfully reaches each blue point, where the *S* matrix is measured. Crucially, these checkpoints are chosen to zigzag about the S-shaped path so that the last three consecutive measurements contain optimal information on the gradient of the *S* matrix with respect to the ball coordinates. **b**, Net momentum imparted to the ball at three different times on the path (black arrow), and its decomposition over the modes injected from the two sides. Note that these were not derived from the ball dynamics but rather inferred from the momentum expectation value of the injected Wigner–Smith eigenstates. Remarkable agreement with the actual direction of the ball velocity, reported in **a** (black arrows), is observed. See Supplementary Video 1. Source data

Figure 2a demonstrates the successful guiding of an object within a disordered medium using acoustic-wave-momentum shaping. Several snapshots of the moving ball are blended into one picture to illustrate better the path followed by the moving scatterer. Supplementary Video 1 (ref. ^49^) was recorded by our camera. Remarkably, the acoustic fields injected from the far field can continuously move the floating ball through a chosen S-shaped trajectory within the disordered medium (the total path length is around four *λ*_0_). Note that the path is discretized into intermediate checkpoints (blue discs) arranged in a zigzag manner about the S-shaped trajectory to enable a good estimation of the *S* matrix gradient (Supplementary Information Section 1.6 and Supplementary Fig. 6). Note that the object is not trapped but moved by successive acoustic pushes, much like a hockey player guiding a puck.

To illustrate the contribution of each mode and in which sense the input state at each time step is optimal, Fig. 2b compares at three distinct times the momentum expectation value of the input superposition (black arrow) with those of the components of its individual modes alone (coloured arrows). It is clear that each mode contributes to pushing the ball in the correct final direction, and the total push is due to the collective action of all modes. Some modes do not push the ball precisely in the desired direction. Yet, this mixture is optimal given the constraints on the wave spatial degrees of freedom imposed by the disordered medium at this specific location. We also compare the total momentum expectation (black arrows in Fig. 2b), which is a theoretical prediction, to the actual velocity of the ball (black arrows in Fig. 2a), which is an experimental observation. The remarkable agreement between the expected momentum push and the measured velocity directions confirms that we successfully implemented our wave-momentum shaping strategy. We conclude that the unavoidable absorption losses present in any experiment, which alter the field amplitudes more than their phases, do not significantly influence the direction of the momentum push predicted by the unitary theory. The interested reader will find other path instances in Supplementary Video 2 (ref. ^49^).

## Angular-momentum transfer

**Fig. 3:**
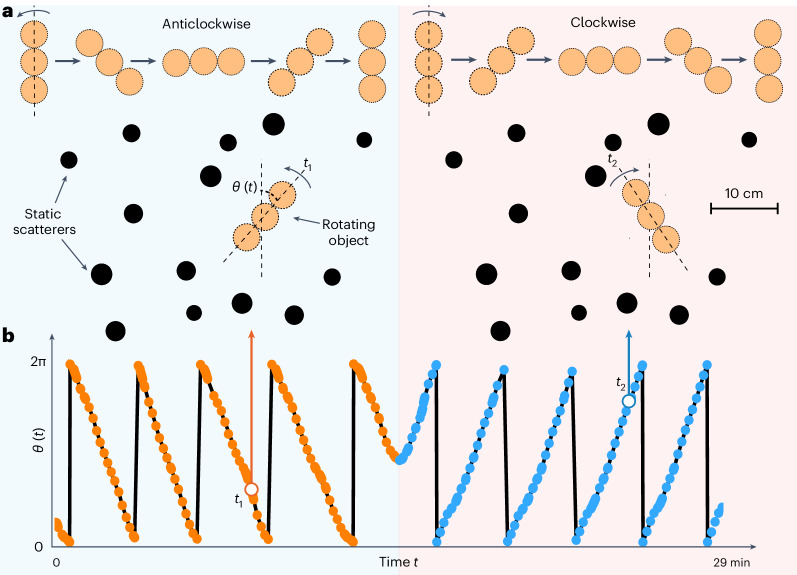
Experimental demonstration of object rotation by acoustic angular-momentum shaping in a static scattering medium. **a**, We use audible sound to rotate an object constructed from three balls glued together in a disordered medium. First, we move the target in the anticlockwise direction (left part of the figure) and then abruptly switch its direction of rotation (right part). At each step (20°), we extract from a far-field *S* matrix measurement the Wigner–Smith operator with respect to the rotation angle *θ*, which allows us to send the wavefront with maximal angular-momentum transfer. **b**, Measured angle versus time, confirming the rotation of the object in the anticlockwise then clockwise directions. Source data

An advantage of the variational principle presented above is that *α* is not restricted to the *x* or *y* coordinate but can be any observable target parameter influencing the scattering. A relevant example we consider in the following is the rotation angle *θ* of an object. This choice will allow us to create an acoustic motor and rotate objects from a distance by sending audible sound. Consider the angular momentum transferred to a rotating object constructed from three balls glued together. Its centre is the rotation axis, which is fixed within the disordered medium (Fig. 3a). The instantaneous scattering matrix *S*(*t*_*m*_) is measured at consecutive time instances *t*_*m*_ with 20° angle step to harness the angular-momentum operator *Q*_*θ*_ and provide a way to induce the optimal transfer of torque from the field to the object. Figure 3b reports the experimentally measured value of *θ* as a function of time. In this experiment, we first selected eigenvectors of *Q*_*θ*_ with positive eigenvalues, consistent with the anticlockwise rotation initially observed during the experiment (blue shaded part of the figure). Then, we abruptly switched to input states with negative eigenvalues (red shaded part). The observation of a reversal of the rotation direction, reported in Fig. 3b, is, thus, consistent with theoretical expectations (Supplementary Video 3 (ref. ^49^)).

## Manipulating in dynamic disorder

**Fig. 4:**
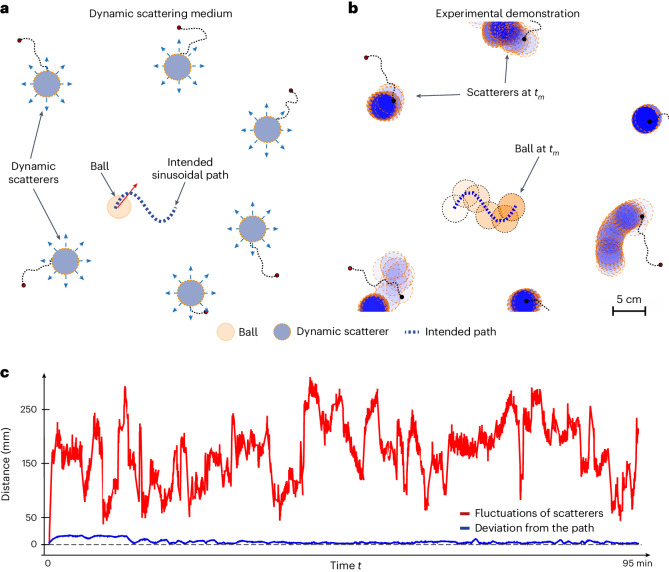
Moving a specific object among a dynamic ensemble of scatterers experiencing random motion. **a**, We let all scatterers move freely under time-dependent external perturbations and wish to control the trajectory of the orange ball, guiding it on a sinusoidal path. The blue balls have a metallic nut glued to them, allowing us to randomize their motion by applying fast magnetic perturbations with a moving external electromagnet. They are loosely anchored to the ground by strings to avoid colliding with the orange ball. **b**, Experimental trajectories measured by a camera, demonstrating the successful control of the ball’s trajectory even in this extreme dynamic scenario. **c**, Comparison between the measured deviation of the target ball centre from the intended sinusoidal path (blue), and the large fluctuations of the other scatterers from their initial positions (red). Source data

As our manipulation method is based on real-time measurements of the instantaneous scattering matrix, the scattering environment can change in time as well. To demonstrate this, we added other floating balls (like the moving target ball) inside the cavity. This experiment and its goal are illustrated in Fig. 4a. The ball we want to control is the orange one. The blue ones are the added balls, which are anchored with light strings to prevent them from colliding with the target ball. The blue balls carry small metallic nuts, which allows us to randomize their motion by varying in time the magnetic field inside the cavity. We wish to control the path of the orange ball and make it follow a shape that looks like a period of a sine function (dashed blue line). Figure 4b shows the successive measured positions of all balls during the experiment. Unlike the blue balls, which move unpredictably, the orange ball closely follows the predesigned sinusoidal path. The deviations of the orange ball from this target path, shown as a blue line in Fig. 4c, are tiny. For comparison, we also plot as a red line the average distance browsed by the blue balls away from their initial position, which fluctuates more strongly in magnitude and speed, underlining the extreme control that we can maintain on the trajectory of the target (details of the different timescales of the dynamic media are discussed in Supplementary Information Section 8). Supplementary Video 4 (ref. ^49^) shows the robustness of the manipulation in the dynamically changing random medium in Fig. 2c .

## Acoustic pressure field maps

**Fig. 5:**
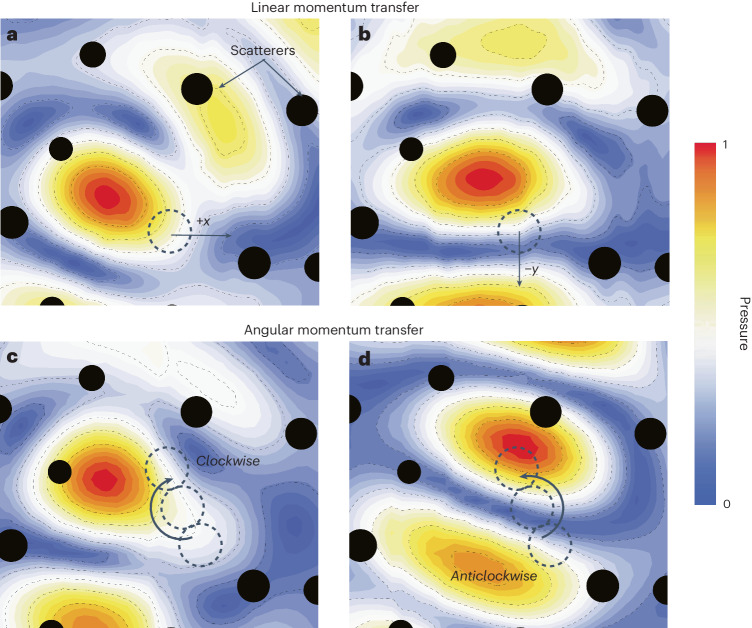
Experimental scans of the field around the moving object. **a**,**b**, Measured distribution of acoustic pressure amplitudes associated with the optimal transfer of linear momentum along the *x* direction (**a**) and the −*y* direction (**b**). **c**,**d**, The rotating object is illustrated for clockwise (**c**) and anticlockwise rotations (**d**). The cylindrical scatterers are represented by black discs, and the moving object is outlined with dashed circles. With our approach, we can create the optimal speckle field allowed by the scattering medium. We automatically create the best possible hot spot next to the object to achieve each prescribed momentum kick.

We provide another point of view on wave-momentum shaping by probing the acoustic pressure field in the vicinity of the moving object (Methods). For this purpose, we exert the optimal linear-momentum push on the ball along different directions, such as +*x* or −*y*, and make it rotate in opposite directions. The acoustic field map is measured. From the displayed pressure profiles (Fig. 5a–d), we see that the speckle field tends to create hot spots of acoustic pressure that push the object in the right direction. Conversely, experimental pressure distributions for the eigenvalues with the smallest absolute value of the corresponding Wigner–Smith operator exhibit no hot spot near the particle and tend to put it in a silent zone (Supplementary Fig. 8). Note that input states that are optimal for motion along *x* can still exhibit a non-zero expectation value in the *y* direction. However, they remain the most efficient at pushing along *x*. Therefore, combining eigenvectors to control expectation values in unwanted directions may provide a way to further refine the algorithm and the precision of the motion. Sometimes the object is in a location in the medium where it cannot be pushed in the right direction given the available degrees of freedom in the speckle. This is not a problem as these points are isolated, and the algorithm will make the object catch up with the trajectory at the next points. To conclude, it is striking to observe how the optimal pressure field can be prepared around the object without knowing anything about the wave–matter interaction involved nor about the object’s shape or environment. This is a clear advantage of the present method compared to conventional methods based on trapping.

## Conclusion

In this Article, we report on the experimental control of an object’s translation and rotation in a complex and dynamic scattering medium using wave-momentum shaping. An iterative manipulation protocol, based solely on knowledge about the far-field scattering matrix of the system and a position guide star, enables the optimal transfer of linear and angular momentum from an acoustic field to manipulate an object in both static and dynamic disordered media. The dynamically injected wavefronts generate the optimal field speckle near the object so that it moves, much like a hockey player guiding a puck, through successive momentum kicks. This method is free of potential traps, robust against disorder and is tolerant of changes in time to the surrounding medium throughout the manipulation. Remarkably, the method is rooted in momentum conservation and does not require any knowledge of the object being manipulated, but only a guide-star measurement of its position. In addition, it does not require any modelling of interaction forces, making the protocol very general and broadly applicable to many real-life scenarios (including different waves, scales, objects and so on). Future efforts will focus on developing methods for objects of various sizes, for example, by transposing the method to ultrasonic frequencies to manipulate smaller objects, as well as extensions to the control of several objects. For this, note that the frequency degree of freedom could also be leveraged. An extension to three-dimensional manipulation would require adequate adaptations, such as a three-dimensional cavity, extraction of three-dimensional coordinates and gradients, and an increased number of channels. Although this seems like a promising direction that would enable motion in a fluid without support for applications in microrobotics, two-dimensional manipulation is already suitable for a vast variety of micromanipulation and acoustofluidic applications in which controlling two of the object’s coordinates is sufficient, for example, tissue engineering^50,51^, drug delivery^52,53^ and biological analysis under a microscope^12^, among others.

## Methods

### Experimental set-up

The set-up consists of a water-filled tank (Fig. 1a and Supplementary Fig. 1a) coupled at the top to a two-dimensional air waveguide terminated at both ends by anechoic terminations. The water tank’s width, length and height are 100 × 100 × 3 cm, respectively. The two-dimensional acoustic waveguide above it has a width of 104 cm, a length of 180 cm and a height of 8 cm. Two columns of ten microphones (ICP 130F20, 1/4 inch, PCB Piezotronics) separated by 5 cm were placed between the tank and the anechoic terminations on each side to measure the distribution of the complex pressure field inside the waveguide. Two columns of ten amplified loudspeakers (Monacor MSH-115, 4 inches, with in-house amplifiers) were placed horizontally on the bottom of each end of the air waveguide to ensure the efficient excitation of all ten modes inside the acoustic structure. The incident wave states were generated with a Speedgoat Performance Real-Time Target Machine controller (I/O 135, sampling rate 10 kHz) with 40 inputs and 20 outputs. The same equipment was used to acquire the corresponding far-field scattering. To generate the proper acoustic wavefronts, the controller was programmed by Matlab/Simulink to control the voltage of the 20 loudspeakers. The voltages produced by 40 microphones corresponding to the pressure signals were then acquired. Sensor conditioners (483C05, PCB Piezotronics) were used to precondition the microphone signals. Examples of captured signals and post-processing are shown in Supplementary Fig. 2.

The moving target scatterer was a ping-pong ball (diameter 4 cm and weight 4.17 g) floating on the water’s surface. The static disorder scatterers were plastic cylinders of various diameters (2 to 4 cm) immersed in the water tank. They extended above the surface but without reaching the upper part of the two-dimensional air waveguide. The cylinders and the ball were coated with candle wax (a hydrophobic film) to prevent the ball from sticking to the static scatterers by capillarity. The real-time position of the ball (Fig. 4) was captured by an ultra-wide webcam (Logitech Brio), working in full high-definition resolution (1,920 × 1,080 pixels) at a high refresh rate (60 frames per second). A small iron nut was glued to the moving target. It was placed at its initial (starting) position by using an electromagnet attached to a mechanical arm (Supplementary Fig. 1b), which was moved in a volume (1,000 × 1,000 × 110 mm) above the water tank by three high-precision linear stages (Newport IMS stages with displacement error <0.05 mm).

The rotating object shown in Fig. 3 comprised three ping-pong balls glued together in a line. A needle was fixed to the bottom of the tank, and the centre of the middle ball was impaled on the needle to prevent linear displacement of the object and allow only rotation while limiting friction. The three balls were painted with different patterns to facilitate the detection of the instantaneous angle.

To create the dynamic scattering medium, we used ten ping-pong balls and glued small metallic nuts onto them. The scattering balls, evenly positioned around the intended paths, were attached to the bottom of the water tank by 3- to 8-cm-long nylon threads. The random fluctuations of these scatterers were exacerbated by randomly moving the mechanical arm over the balls while randomly switching the state of the attached electromagnet. The disorder scatterers were placed at a significant enough distance from the target scatterer, which was still free-floating, to avoid any collisions.

Finally, the top plates above the water tank could be replaced with carefully designed perforated plates (holes with a diameter of 1 mm and forming a square array with a period of 10 mm; Supplementary Fig. 1e) to allow the field inside the waveguide to be scanned by a microphone placed on the robotic arm outside the waveguide.

### Scattering matrix measurement

The complex scattering matrix *S* relates the incoming with the outgoing flux-normalized modes through a set of 2*N* linearly independent equations (2*N* = 20 is the total number of propagative modes, ten from each side): **ψ**_out_ = *S* **ψ**_in_. Solving the scattering matrix *S* requires measuring *N* independent wave mode distributions excited by a combination of speakers that form an orthogonal basis. Our experiment uses an orthonormal basis, and only one speaker is excited at a time with the 1,590 Hz harmonic signal. For each excitation, the data collected by the microphone arrays on both sides can be used to determine the incident and outgoing modes **ψ**_in,out_. With the hardware we used, this takes about 80 ms. Therefore, after 2*N* orthogonal excitations (1.6 s), the scattering matrix is solved for that particular scattering configuration. This type of raw scattering matrix is neither perfectly symmetric nor unitary and is subsequently regularized by discarding its very small antisymmetric part and rescaling its subunitary eigenvalues while keeping their phases (Supplementary Fig. 3).

### Construction of GWS operators

The construction of the GWS operator *Q*_*α*_ is based on gradient approximations, which require successive measurements of the scattering matrix *S*(*t*) at three different positions (time).

To derive the translation GWS operators *Q*_*x*_ and *Q*_*y*_ for the ball at position (*x*_*m*_, *y*_*m*_) and time instance *t*_*m*_, we need, in addition to the scattering matrix *S*_*m*_ measured at the actual position, the scattering matrices *S*_*m*−1_ and *S*_*m*−2_ measured at the two previous time instances *t*_*m*−1_ and *t*_*m*−2_, when the ball was at coordinates (*x*_*m*−1_, *y*_*m*−1_) and (*x*_*m*−2_, *y*_*m*−2_), respectively.

With these three matrices *S*_*m*_, *S*_*m*−1_ and *S*_*m*−2_, the gradient of *S* can be derived with the following approximation formula:

3 $$\left[\begin{array}{c}{\partial}_{x}{{{S}}}_{m}\\ {\partial}_{y}{{{S}}}_{m}\end{array}\right]\approx {\left[\begin{array}{cc}{x}_{m}-{x}_{m-2}&{y}_{m}-{y}_{m-2}\\ {x}_{m}-{x}_{m-1}&{y}_{m}-{y}_{m-1}\end{array}\right]}^{-1}\cdot \left[\begin{array}{c}{{{S}}}_{m}-{{{S}}}_{m-2}\\ {{{S}}}_{m}-{{{S}}}_{m-1}\end{array}\right].$$

With the gradient estimated, the construction of the GWS operators *Q*_*x*_ and *Q*_*y*_ is direct:

$$\begin{aligned}{{{Q}}}_{x}&=-{{{\rm{i}}}}{{{S}}}_{m}^{-1}\,{\partial}_{x}{{{S}}}_{m},\\ {{{Q}}}_{y}&=-{{{\rm{i}}}}{{{S}}}_{m}^{-1}\,{\partial}_{y}{{{S}}}_{m}.\end{aligned}$$

The error in the gradient approximation and, therefore, in the operators *Q*_*x*_ and *Q*_*y*_, depends on the shape of the triangle formed by the three measurement points, with the best results obtained for an equilateral triangle and worse for a flat scalene one (Supplementary Fig. 6). A detailed analysis of the triangle’s influence on the derivation of the GWS operator is provided in Supplementary Information Section 6. Therefore, for the best manipulation of the object position, the path is drawn with a zigzag line to minimize the error in the GWS operators.

Similarly, the rotation GWS operator *Q*_*θ*_ requires the measurement of *S*_*m*_, *S*_*m*−1_ and *S*_*m*−2_ for three consecutive vane angles *θ*_*m*_, *θ*_*m*−1_ and *θ*_*m*−2_ taken at times *t*_*m*_, *t*_*m*−1_ and *t*_*m*−2_.

The gradient approximation is, in that case, derived with a backward three-point derivative

4 $${\partial}_{\theta}{{{S}}}_{m}\approx \frac{{{{S}}}_{m}-4{{{S}}}_{m-1}+3{{{S}}}_{m-2}}{\updelta \theta},$$

where δ*θ* is the angle difference between the time instances *t*_*m*_ and *t*_*m*−1_.

The GWS operator constructed to control the rotation of the vanes, therefore, reads as follows

5 $${{{Q}}}_{\theta}=-{{{\rm{i}}}}{{{S}}}_{m}^{-1}\,{\partial}_{\theta}{{{S}}}_{m}.$$

### Injection of optimal input mode mixtures

As explained in the text, finding the optimal mode mixture to be injected to give the optimal momentum push to the object follows from equation (1). We, therefore, provide a short proof for this important equation.

For a particle in free space, the change of momentum transferred to it upon scattering Δ*p*_*α*_ can be calculated from the expectation values of the operator *C*_*α*_ = −i∂/∂*α* for the superposition states $\left\vert {{{{\boldsymbol{\Psi }}}}}_\mathrm{in,out}\right\rangle$

6 $$\Delta {p}_{\alpha }=\left\langle {{{{\boldsymbol{\Psi }}}}}_{{{{\mathrm{out}}}}}| C_{\alpha }| {{{{\boldsymbol{\Psi }}}}}_{{{{\mathrm{out}}}}}\right\rangle -\left\langle {{{{\boldsymbol{\Psi }}}}}_{{{{\mathrm{in}}}}}| C_{\alpha }| {{{{\boldsymbol{\Psi }}}}}_{{{{\mathrm{in}}}}}\right\rangle ,$$

We can then write:

7 $$\Delta {p}_{\alpha}=-\mathrm{i}\left(\left\langle {{{{\boldsymbol{\Psi}}}}}_{{{\mathrm{out}}}}\middle| \frac{\mathrm{d}}{\mathrm{d}\alpha}\middle| {{{{\boldsymbol{\Psi}}}}}_{{{\mathrm{out}}}}\right\rangle -\left\langle {{{{\boldsymbol{\Psi}}}}}_{{{\mathrm{in}}}}\middle| \frac{\mathrm{d}}{\mathrm{d}\alpha}\middle| {{{{\boldsymbol{\Psi}}}}}_{{{\mathrm{in}}}}\right\rangle \right).$$

Using *S*^†^*S* = 1, we can write 〈**Ψ**_in_∣d/d*α*∣**Ψ**_in_〉 = 〈*S***Ψ**_in_∣*S* d/d*α*∣**Ψ**_in_〉 and obtain

8 $$\Delta {p}_{\alpha}=-\mathrm{i}\left\langle {{S}}{{{{\boldsymbol{\Psi}}}}}_{{{\mathrm{in}}}}\vert \left(\left.\frac{\mathrm{d}}{\mathrm{d}\alpha}\vert {{S}}{{{{\boldsymbol{\Psi}}}}}_{{{\mathrm{in}}}}\right\rangle -{{S}}\frac{\mathrm{d}}{\mathrm{d}\alpha}\vert {{{{\boldsymbol{\Psi}}}}}_{{{\mathrm{in}}}}\right\rangle \right).$$

The term in parentheses is nothing but $\frac{\mathrm{d}S}{\mathrm{d}\alpha}\left\vert\mathbf{\Psi}_{{{{\mathrm{in}}}}}\right\rangle$, and we directly get

9 $$\Delta {p}_{\alpha}=-\mathrm{i}\left\langle {{S}}{{{{\boldsymbol{\Psi}}}}}_{{{\mathrm{in}}}}\middle| \frac{\mathrm{d}S}{\mathrm{d}\alpha}\middle| {{{{\boldsymbol{\Psi}}}}}_{{{\mathrm{in}}}}\right\rangle =\left\langle {{{{\boldsymbol{\Psi}}}}}_{{{\mathrm{in}}}}\middle| -\mathrm{i}{{{S}}}^{{\dagger}}\frac{\mathrm{d}S}{\mathrm{d}\alpha}\middle| {{{{\boldsymbol{\Psi}}}}}_{{{\mathrm{in}}}}\right\rangle .$$

In refs. ^44,45^, it was shown that equation (1) continues to hold, even when the target particle is embedded in a scattering environment. In this way, the momentum push expected for a given far-field input is expressed as the expectation value of the GWS operator *Q*_*α*_, which is Hermitian. Therefore, the eigenvector of *Q*_*α*_ provides the optimal momentum push with the highest eigenvalue.

Having measured the Wigner–Smith operators, we diagonalize them and find the eigenvector with the highest eigenvalue. We use the eigenvalues to calculate the optimal mode mixture to be injected to give the optimal momentum push to the particle. For example, if we want to move the object by Δ*x* and Δ*y*: (1) We diagonalize *Q*_*x*_ and *Q*_*y*_. (2) We obtain their eigenvectors with highest eigenvalues **Ψ**_*x*,*y*_, with eigenvalues calculated as δ*x* and δ*y*. (3) We construct the optimal input state $\frac{\Delta\, x}{\delta x}{\mathbf{\Psi}}_{x}+\frac{\Delta\, y}{\delta y}{\mathbf{\Psi}}_{y}$. This input state is multiplied by the coupling coefficient matrix of the speakers *M* to give the required voltage amplitudes and phases required on each speaker (Supplementary Figs. 2 and 5). In practice, to determine the direction we want to go, we measure the position of the ball (*x*, *y*) at a given time (Supplementary Fig. 4) and compare it with the position of the next checkpoint on the trajectory, which we try to reach up to a certain threshold distance before moving on to the next checkpoint.

## Online content

Any methods, additional references, Nature Portfolio reporting summaries, source data, extended data, supplementary information, acknowledgements, peer review information; details of author contributions and competing interests; and statements of data and code availability are available at 10.1038/s41567-024-02538-5.

## Supplementary information

Supplementary Information Supplementary Text and Figs. 1–9.

Supplementary Video 1 Experimental video demonstrating object manipulation in disordered medium along the long path.

Supplementary Video 2 Experimental video demonstrating object manipulation in disordered medium along the different trajectories.

Supplementary Video 3 Experimental video demonstrating object rotation in a disordered medium.

Supplementary Video 4 Experimental video demonstrating the ability to manipulate the object in a dynamic disordered medium.

## Source data

Source Data Fig. 1 Source data for *S* matrices.

Source Data Fig. 2 Source data for the path points and vectors.

Source Data Fig. 3 Source data for rotation angles and scatterer positions.

Source Data Fig. 4 Source data for ball deviation and fluctuations of medium.

## Data Availability

Source data are provided with this paper. These data are also available via Zenodo at 10.5281/zenodo.10207638 (ref. ^49^). All other data that support the plots within this paper and other findings of this study are available from the corresponding author upon reasonable request.

## Code availability

The codes that support the findings of this study are available from the authors upon reasonable request.
