# Supplementary material for: Wave-momentum shaping for moving objects in heterogeneous and dynamic media
Source: Nat Phys. 2024 Jun 21;20(9):1441–7. doi: 10.1038/s41567-024-02538-5 (PMC11392811; doi:10.1038/s41567-024-02538-5)
Supplement: Supplementary file 1 — Supplementary Text and Figs. 1–9. [file 41567_2024_2538_MOESM1_ESM.pdf]

nature physics

Supplementary information https://doi.org/10.1038/s41567-024-02538-5

Wave-momentum shaping for moving objects in heterogeneous and dynamic media

In the format provided by the authors and unedited

Contents

1 Extraction of scattering matrices 2

2 Signal processing and excitation of the wave state 3

3 Regularization method for the measured scattering matrices 4

4 Detection and Localization of Target 4

5 Constructing Wigner-Smith operators by utilizing the S gradient approximation 5

6 Angular dependence of gradient approximation accuracy 6

7 Target manipulation algorithm and control design 6

8 Dynamic medium and processing time 7

1 Extraction of scattering matrices

We aim to control the target's position inside the disordered medium by analyzing the far field scattering, represented by the scattering matrix $\boldsymbol{S}$. We start by considering the set-up as a 2D acoustic waveguide carrying N propagative modes (see Fig. S1). The waveguide is excited by a harmonic wave of pulsation ω, $\Xi(x,y,t) = \Psi(x,y)\mathrm{e}^{\mathrm{i}\omega t}$ which follows the Helmholtz equation

$$\Delta\Psi(x,y) + k_0^2\Psi(x,y) = 0, \tag{A.1}$$

where k_0 is the wavenumber.

Far from the central part where scattering occurs, the acoustic field can be decomposed into the transverse mode basis ϕ_n, satisfying orthogonality

$$\int_0^1 \phi_n(y)\phi_m(y)dy = \delta_{nm}, \tag{A.2}$$

where δ_{nm} is the Kronecker symbol. The modes are determined from the boundary conditions in the transverse direction, in this case, Neumann boundary condition at $y = 0, L$, and read as follows

$$\phi_n = \sqrt{\frac{2}{D}}\cos(\frac{n\pi}{D}y). \tag{A.3}$$

Each transverse function is associated with a specific propagation constant (eigenvalue) $k_n^2 = k_0^2 - (n\pi/D)^2$. The modes are propagative if k_n is real, i.e., $k_0^2 > (n\pi/D)^2$. For a 1590 Hz excitation in the present waveguide, we have $N = \omega D/c\pi \approx 10$ propagative modes. The higher modes are evanescent.

For each mode n, the acoustic field can be described as a linear superposition of incoming and outgoing waves of amplitudes $\psi_n^{\pm}$.

$$\Psi(x,y) \approx \sum_{n=0}^{N-1}\Psi_n(x,y) \approx \sum_{n=0}^{N-1}\left(\psi_n^+\mathrm{e}^{-\mathrm{i}k_n x} + \psi_n^-\mathrm{e}^{\mathrm{i}k_n x}\right)\phi_n(y). \tag{A.4}$$

On each side of the scattering region, denoted by the upper script (1) or (2) for the left and right sides, respectively, we use two columns of 10 microphones to distinguish between forward and backward propagating waves (see Fig. S1), respectively $\boldsymbol{\psi}_{\mathbf{l}}^{(\mathbf{1,2})^+} = \{\psi_{l,0}^{(1,2)+}, \cdots, \psi_{l,N}^{(1,2)+}\}^T$ and $\boldsymbol{\psi}_{\mathbf{l}}^{(\mathbf{1,2})^-} = \{\psi_{l,0}^{(1,2)-}, \cdots, \psi_{l,N}^{(1,2)-}\}^T$.

For the microphone pairs in the row l, the total pressures $p_{m_{l,1}}$ and $p_{m_{l,2}}$ (resp. $p_{m_{l,3}}$ and $p_{m_{l,4}}$) at position x_1 and x_2 (resp. x_3 and x_4) are related to $\psi_{l,n}^{(1)+}$ and $\psi_{l,n}^{(1)-}$ (resp. $\psi_{l,n}^{(2)+}$ and $\psi_{l,n}^{(2)-}$) for each propagating constant $k_n = \omega_n/c$ as

$$\left[\psi_{l,n}^{(1)+}\ \psi_{l,n}^{(1)-}\right] = \left[p_{m_{l,1}}(\omega_n)\ p_{m_{l,2}}(\omega_n)\right]\cdot\begin{bmatrix}\mathrm{e}^{-\mathrm{i}k_n x_1} & \mathrm{e}^{-\mathrm{i}k_n x_2}\\ \mathrm{e}^{\mathrm{i}k_n x_1} & \mathrm{e}^{\mathrm{i}k_n x_2}\end{bmatrix}^{-1}. \tag{A.5}$$

The scattering matrix $\boldsymbol{S}$ which relates the waves incoming $\boldsymbol{\psi}_{\mathbf{l,in}}$ and outgoing $\boldsymbol{\psi}_{\mathbf{l,out}}$ from the scattering region to be characterized reads as follows

$$\boldsymbol{\psi}_{\mathbf{l,out}} = \begin{pmatrix} \boldsymbol{\psi}_{\mathbf{l}}^{(\mathbf{1})^-} \\ \boldsymbol{\psi}_{\mathbf{l}}^{(\mathbf{2})^+} \end{pmatrix} = \begin{bmatrix} \mathbf{r}^{(\mathbf{1})} & \mathbf{t}^{(\mathbf{2})} \\ \mathbf{t}^{(\mathbf{1})} & \mathbf{r}^{(\mathbf{2})} \end{bmatrix} \cdot \begin{pmatrix} \boldsymbol{\psi}_{\mathbf{l}}^{(\mathbf{1})^+} \\ \boldsymbol{\psi}_{\mathbf{l}}^{(\mathbf{2})^-} \end{pmatrix} = \boldsymbol{S} \cdot \boldsymbol{\psi}_{\mathbf{l,in}}, \tag{A.6}$$

with $\mathbf{r}^{(\mathbf{1,2})}$ and $\mathbf{t}^{(\mathbf{1,2})}$ the N by N reflection and transmission matrices for ports (1) and (2) respectively.

The scattering, reflection, and transmission matrices characterize the whole system, so eq. (A.6) is unique and valid for the $\boldsymbol{\psi}_{\mathbf{in/out}}$ states determined by any of the 10 microphone pairs. It can be rewritten

$$\left[\boldsymbol{\psi}_{\mathbf{1,out}} \cdots \boldsymbol{\psi}_{\mathbf{10,out}}\right] = \boldsymbol{S} \cdot \left[\boldsymbol{\psi}_{\mathbf{1,in}} \cdots \boldsymbol{\psi}_{\mathbf{10,in}}\right]. \tag{A.7}$$

The extraction of the scattering matrix can then be performed by successively inverting the submatrices of eq. (A.7)

$$\mathbf{r}^{(\mathbf{1})} = \left[\boldsymbol{\psi}_{\mathbf{1}}^{(\mathbf{1})^-} \dots \boldsymbol{\psi}_{\mathbf{10}}^{(\mathbf{1})^-}\right] \cdot \left[\boldsymbol{\psi}_{\mathbf{1}}^{(\mathbf{1})^+} \dots \boldsymbol{\psi}_{\mathbf{10}}^{(\mathbf{1})^+}\right]^{-1} \tag{A.8}$$

$$\mathbf{t}^{(\mathbf{1})} = \left[\boldsymbol{\psi}_{\mathbf{1}}^{(\mathbf{2})^+} \dots \boldsymbol{\psi}_{\mathbf{10}}^{(\mathbf{2})^+}\right] \cdot \left[\boldsymbol{\psi}_{\mathbf{1}}^{(\mathbf{1})^+} \dots \boldsymbol{\psi}_{\mathbf{10}}^{(\mathbf{1})^+}\right]^{-1} \tag{A.9}$$

$$\mathbf{t}^{(\mathbf{2})} = \left[\psi_{\mathbf{1}}^{(\mathbf{1})^-} \dots \boldsymbol{\psi}_{\mathbf{10}}^{(\mathbf{1})^-}\right] \cdot \left[\boldsymbol{\psi}_{\mathbf{1}}^{(\mathbf{2})^-} \dots \boldsymbol{\psi}_{\mathbf{10}}^{(\mathbf{2})^-}\right]^{-1} \tag{A.10}$$

$$\mathbf{r}^{(\mathbf{2})} = \left[\boldsymbol{\psi}_{\mathbf{1}}^{(\mathbf{2})^+} \dots \boldsymbol{\psi}_{\mathbf{10}}^{(\mathbf{2})^+}\right] \cdot \left[\boldsymbol{\psi}_{\mathbf{1}}^{(\mathbf{2})^-} \dots \boldsymbol{\psi}_{\mathbf{10}}^{(\mathbf{2})^-}\right]^{-1}. \tag{A.11}$$

2 Signal processing and excitation of the wave state

To characterize the scattering within the acoustic waveguide, we use the experimental setup described in Methods and illustrated by the photograph in Fig. S1. The extraction of the scattering matrix is performed in a step-by-step experimental procedure. Each of the 20 speakers (marked by the black disks in Fig. S1 **a**) is excited sequentially by a sinusoidal wave of frequency $f_0 = 1590$ Hz to form an orthonormal basis of wave states as explained in Methods. The excitation duration for each speaker is 0.08 s, with a total excitation time of 1.6 s. As an example, Fig. S2 **a** (top inset) exhibits the recorded voltage signal from microphone $m_{8,2}$ for all consecutive speaker excitations while the bottom inset focuses specifically on the excitation of speaker 18th. The magnitude and phase of the signal are determined through a Fourier transform, as depicted in Figs. S2 **b**, upper and lower insets, respectively. The measured magnitude and phase on the 4 rows of 10 microphones are concatenated in Fig. S2 **c**.

Using eq. (A.5), both in-going $\boldsymbol{\psi}_{\mathbf{in}}$ and out-going $\boldsymbol{\psi}_{\mathbf{out}}$ states can be measured from both sides of the scattering region. Ultimately, the solution to Eq. (A.7) allows for the derivation of the scattering matrix $\boldsymbol{S}$.

Moreover, to properly characterize and excite the desired wavefront within the cavity, it is necessary to establish an accurate relationship between excitation voltages, $\mathbf{U_{exc}}$, and input wave states $\boldsymbol{\psi}_{\mathbf{in}}$ in the system. To do so, we compute the coupling coefficient matrix $\boldsymbol{M} = \mathbf{U_{exc}}/\boldsymbol{\psi}_{\mathbf{in}}$ shown in Fig. S2 **d**. The output signals are physically synchronized with the inputs using BNC cables to address potential phase discrepancies stemming from synchronization delays within the Speedgoat controller. That way, we can correctly determine, from the eigenvectors determined from the WS operator, the loudspeaker voltages (in terms of amplitude and phase) required to excite any waveguide input state based on a specified scattering configuration (represented by the $\boldsymbol{S}$ matrix).

For the voltages shown in Fig. S2 during the **S** matrix extraction, the corresponding maximum peak pressure reached values of $p_{max} \approx 5$ Pa. On the other hand, the pressures inside of the waveguide can reach values as high as $p_{max} \approx 15$ Pa during the optimal wavestate excitation since multiple speakers are excited, as shown by the voltages in Fig. S5.

3 Regularization method for the measured scattering matrices

Due to inherent losses and inherent noise in the measurements, the extracted scattering matrices exhibit sub-unitary behavior and lack perfect symmetry (reciprocity). As a prerequisite for applying the theory, it becomes necessary to impose symmetry and reciprocity on these matrices through subsequent post-processing steps. The first step involves discarding their anti-symmetric components and taking only the symmetric part: $\boldsymbol{S}_{sym} = \frac{1}{2}[\boldsymbol{S} + \boldsymbol{S}^T]$, followed by a subsequent operation that entails determining the eigenvalues of the symmetric portion and then scaling their magnitudes to unity while retaining their phases. The regularization process for the magnitudes is visually demonstrated in Fig. S3 **a,b**, illustrating the eigenvalues e_{ij} before (depicted as circles) and after correction (represented as squares) in scenarios both (**a**) with and (**b**) without the presence of cylindrical scatterers.

4 Detection and Localization of Target

Obtaining the (x, y) coordinates of the object is vital for efficiently manipulating the target and ensuring precise placement. To achieve this objective, a camera beneath the water tank is employed to detect and precisely determine the location of the target ball. The physical coordinates of the scatterer are derived by establishing the origin of axes within a mobile arm (linked to three linear stages of the Newport IMS® series, see Fig. S1) and calibrating the camera using an image of the empty waveguide. Moreover, we use a green screen on the waveguide to enhance the contrast between the scatterers and the background (see Fig. S4 **a**). Before employing a disk detection algorithm in Matlab, each frame is pre-processed using its image processing tools. First, we use the

intensity of the red channel of the image (as shown in Fig. S4 **b**) and apply the contrast enhancement (see Fig. S4 **c**). Then, the circular objects within the waveguide are identified, enabling the capture of high-contrast circles of various radii. The circle corresponding to the target is singled out by filtering out circles with a radius equivalent to that of the ping-pong ball (yellow circles in Fig. S4), yielding an exact real-time determination of the target's position in pixel coordinates. The precision of determination becomes more challenging in the rotation scenario, where the rotation angle of all three white balls with equal radii is sought, as depicted by the yellow contours in Fig.S4. To accurately extract the vane scatterer's angle, a black disc is painted beneath one of the exterior white ping-pong balls, serving as a reference point (a green contour indicates the reference ball Fig.S4 **c**). The static black scatterers, which already exhibit superior contrast against the background, are located using a similar approach, depicted by the red discs in Fig.S4 **c**.

5 Constructing Wigner-Smith operators by utilizing the S gradient approximation

After extracting the scattering matrices $\boldsymbol{S}_{m-2}$,$\boldsymbol{S}_{m-1}$, and $\boldsymbol{S}_m$ at three consecutive time instances t_{m-2}, t_{m-1}, and t_m, when the moving object was located respectively at the corresponding three positions (x_{m-2}, y_{m-2}), (x_{m-1}, y_{m-1}), and (x_m, y_m), as shown in Fig. S5 **a**, we can find the gradient of $\boldsymbol{S}$ using the following approximation

$$\begin{bmatrix} \boldsymbol{S}_m - \boldsymbol{S}_{m-2} \\ \boldsymbol{S}_m - \boldsymbol{S}_{m-1} \end{bmatrix} \approx \begin{bmatrix} x_m - x_{m-2} & y_m - y_{m-2} \\ x_m - x_{m-1} & y_m - y_{m-1} \end{bmatrix} \begin{bmatrix} \partial_x \boldsymbol{S}_m \\ \partial_y \boldsymbol{S}_m \end{bmatrix}. \tag{A.12}$$

Therefore, we can construct the WS operators $Q_{x,m}, Q_{y,m}$ for the corresponding displacements in (x, y)

$$\begin{aligned} \boldsymbol{Q}_{x,m} &= -\mathrm{i}\boldsymbol{S}_m^{-1}\partial_x \boldsymbol{S}_m, \\ \boldsymbol{Q}_{y,m} &= -\mathrm{i}\boldsymbol{S}_m^{-1}\partial_y \boldsymbol{S}_m. \end{aligned}$$

Examples of the constructed WS operators $\boldsymbol{Q}_x, \boldsymbol{Q}_y$ are plotted in Fig. S5 **b**.

Diagonalizing $\boldsymbol{Q}_x$ and $\boldsymbol{Q}_y$ gives access to the eigenvalues of both operators. Since the eigenvalue that most efficiently transfers the linear/angular momentum to the target is the highest, we sort the eigenvalues in descending order. We then calculate the eigenvectors of the highest eigenvalues of $\boldsymbol{Q}_x$ and $\boldsymbol{Q}_y$ that correspond to the displacement to be performed, i.e. a positive displacement for ”X” and a negative displacement for ”Y” in the case of the situation in Fig. S5 **a**, as illustrated in Figs. S5 **b,c**.

6 Angular dependence of gradient approximation accuracy

As described in the previous section, the construction of the WS operators relies on a 2D gradient approximation, implying three positions in ”x” and ”y.” With Fig. S6, we investigate the influence of the choice of these three positions on the accuracy of approximating the gradient and, therefore, constructing the WS operators.

We start from the optimal configuration, an equilateral triangle with an angle $\varphi = \pi/3$, and degrade it by reducing the angle to $\varphi = \pi/8$ and $\varphi = \pi/36$, by flatting the triangle. The experimental displacements (filled color discs) and expectation value (Re[$< \psi_{\mathbf{in}} \mid \boldsymbol{Q}_{\mathbf{x},\mathbf{y}} \mid \psi_{\mathbf{in}} >$]) obtained numerically directly from the WS operators for these three configurations are shown in Figs. S6 **a,b,c** respectively.

We successively target for each case a displacement in ”-X” (blue), ”+X” (red), ”-Y” (orange), and ”+Y” (green), and repeat for statistical reason 15 times the measurement. As evidenced, the experimental displacement matches the best with the expectation value when the three positions used for the gradient approximation form an equilateral triangle. The flatter the triangle, the more the error in the approximation and the greater the discrepancies. Based on this observation, the smooth target path the object has to follow is discretized in a zigzag-like motion to improve the gradient approximation.

7 Target manipulation algorithm and control design

We first discretize the target path into a zig-zag-like path with equidistant target points and initialize the system by measuring the $\boldsymbol{S}$ matrices at the first three locations. From this, we start the algorithm, detect the ball’s position, and estimate the distance to the nearest path point. If the distance from the path point is lower than a given tolerance value, the ball has attained a target position. We then check if we already measured $\boldsymbol{S}$ at this position. If this is the case (for example, just after initialization), we directly compute the GWS operator from the three previous scattering matrices. If we don’t have a measure of $\boldsymbol{S}$ at the actual location, we first measure it and then compute the GWS operator. We select the optimal incident state by solving the eigenvalue problem and then generate the selected wavefront during a given time t_p by exciting the 20 speakers with the proper phase and amplitude voltages, to let the ball drift to the next position. If we stop the excitation, the ball eventually stops due to friction. Once the ball position is stable, we start the algorithm again and loop until the whole path is covered.

8 Dynamic medium and processing time

The dynamic environment studied here consists of fifteen other ping-pong balls floating on the water surface which are anchored to the bottom of the water tank by thin strings. The length of the strings (8.5 cm on average) was chosen to allow the disorder to evolve randomly over long distances (fluctuations are hundreds of millimeters in Fig. 4c, a distance comparable to the wavelength) while preventing any collision with the target ball. Small metal nuts were glued to each scatterer to amplify the disorder's movement using a magnetic field, and the latter was intentionally randomly modified by moving an electromagnet with the robotic arm over the cavity.

There are two relevant times in the case of micromanipulation inside a dynamic medium. The first one is the medium correlation time t_{corr}, namely the time it takes for the Wigner-Smith eigenstates to be uncorrelated. This time is related to the speckle correlation time, and depends not only on the medium's fluctuations, but also on the object's trajectory and speed. The second important characteristic time is the processing time, t_{proc}, namely the time it takes for position detection, scattering measurements, WS eigenstate computation, and wavefront excitation. This time is mostly limited by the speed of the electronics used for acquisition and processing, and shortening it is a pure technological problem. When $t_{proc}/t_{corr} \ll 1$, we are in the quasi-static regime, where the method will work nicely. The limit is therefore when $t_{proc}/t_{corr} = 1$, because beyond this point the medium changes faster than we can react. In our experiment, we estimate that $t_{corr} \approx 10 t_{proc}$, by measuring the correlation time of the Wigner-Smith operator. We therefore operate in an intermediate regime that is not quasi-static. It is worth noting that in the majority of biology applications, the dynamics of the medium are slow enough to allow efficient manipulation.

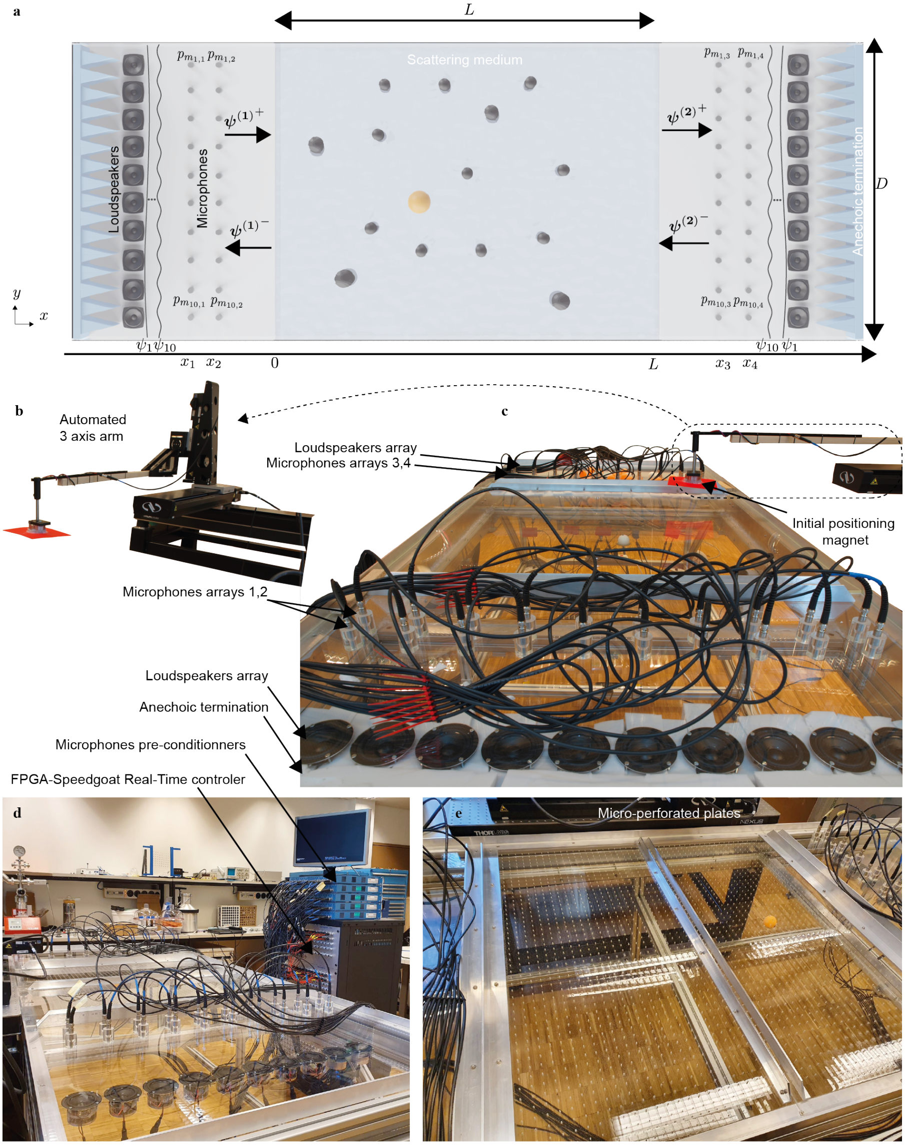

Fig. S1 Experimental set-up details. We operate at a frequency where a parallel plate acoustic waveguide (made of transparent plastic) supports 10 propagating modes. In the middle, the so-called scattering region, the bottom wall of the waveguide is replaced by the water-air interface of a tank. On each side, 10 speakers allow us to control the mode mixture sent to the scattering region. Two rows of 10 microphones on each side allow us to extract the incident and reflected fields corresponding to each mode, allowing the real-time extraction of the scattering matrix. **b** A 3-axis robotic arm is used to automatize the placement of the moving ball. It is also used when performing pressure field scanning, where the top waveguide plate is replaced by a micro-perforated plate (**e**).

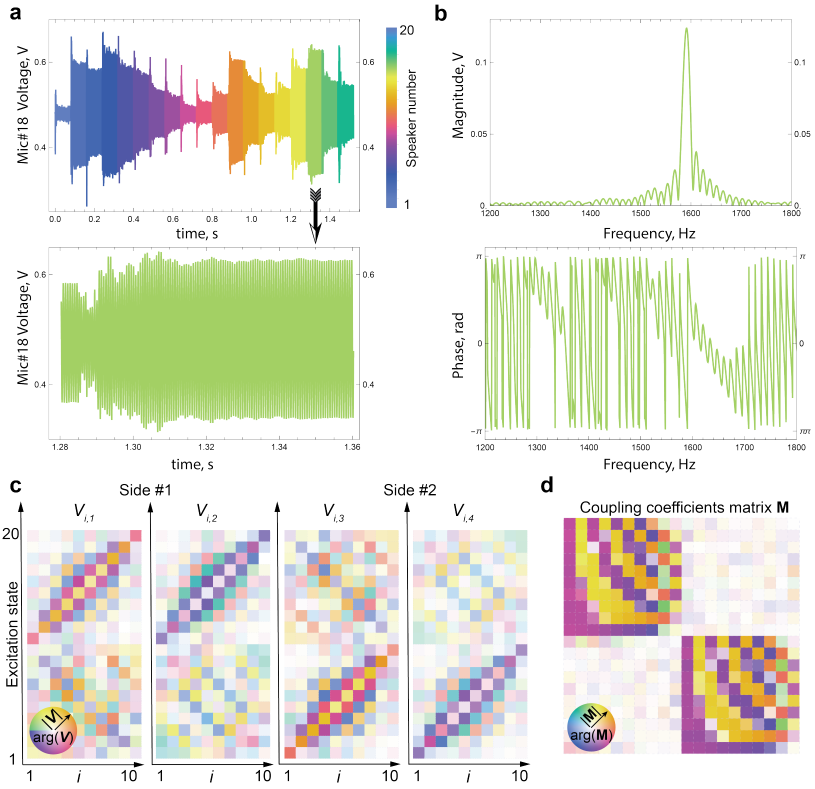

Fig. S2 Captured microphone signals and extraction of waveguide modes. To excite the particular waveguide mode and measure the scattering in the system, we use one column of 10 speakers and two columns of 10 microphones on each side of the scattering region. The scattering in the system is measured by exciting each of the speakers successively with a 1590 Hz sine-wave. **a** shows the voltage measured at microphone $m_{2,8}$ for all the excitation (upper inset) and only the 18th speaker ($m_{8,2}$) (lower inset). The time signal is then Fourier transform to access the magnitude and phase on each microphone pair for all the modes as illustrated in **c**. **d** To be able to excite a specific wavefront in the system, the eigenvectors obtained from the WS operator have to be converted through excitation voltages for each speaker via a coupling coefficient matrix **M** that accounts for the speaker frequency response.

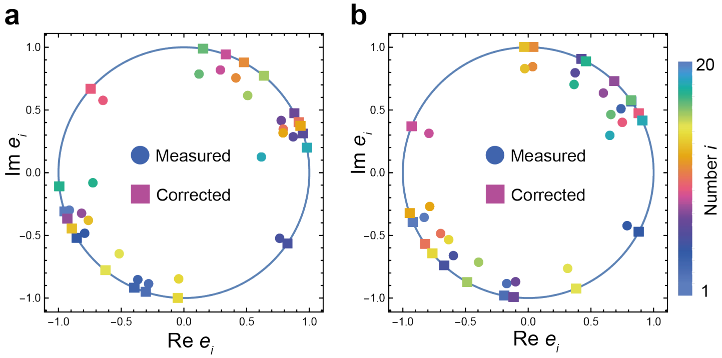

Fig. S3 Regularization method for the measured scattering matrices. Due to losses, the measured scattering matrices are generally sub-unitary. In addition, due to measurement noise, they are not perfectly symmetric (reciprocal). To apply the theory, we first regularize these matrices by neglecting their anti-symmetric part and then regularizing the sub-unitary symmetric part. We rescale their eigenvalues, keeping their phases but setting their moduli to one. Eigenvalues e_{ij} before (circles) and after correction (squares) in the (**a**) absence and (**b**) presence of the cylindrical scatterers.

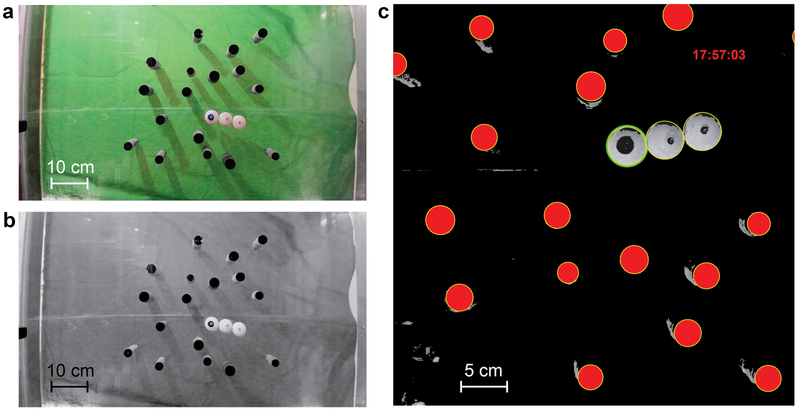

Fig. S4 A standard image captured by the camera depicts the identified rotating object and scatterers. a, The camera's perspective is directed toward the ceiling beneath the transparent waveguide covered with a green screen to enhance the contrast. **b**, The red channel of each frame is used for object detection and recognition since it provides the best contrast. **c**, The intensity of pixels is squared to increase the contrast further and improve the disk recognition. The stationary scatterers are recognized and marked with yellow circles through image processing. The trio of balls comprising the rotating object is detected and indicated by green circles. To distinguish a reference ball and determine the rotation angle, one of the outer balls is marked with a black disk at the bottom.

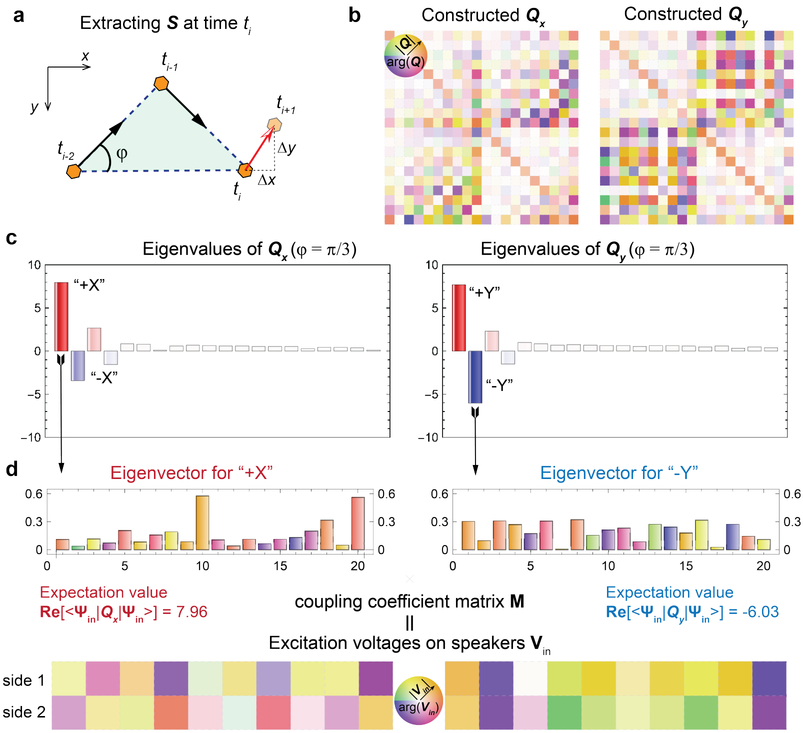

Fig. S5 Extraction of the Wigner-Smith operator from S matrix measurement at three consecutive time-steps. **a**, At the time t_i, we use the S matrix measured at the three consecutive times t_{i-2}, t_{i-1} and t_i, to push the ball towards the next point at t_{i+1}, defined by the increments Δx and Δy. **b** Example of the GWS operators $\mathbf{Q_x}$ (right inset) and $\mathbf{Q_y}$ (left inset) computed from the gradient approximation utilizing the measurements of S at t_{i-2}, t_{i-1} and t_i. **c** Sorted eigenvalues of the operators obtained after diagonalizing $\mathbf{Q_x}$ and $\mathbf{Q_y}$. **d** Computed eigenvectors (upper panels) corresponding to the maximum eigenvalues in the direction of the intended motion following a zigzag trajectory ($\Delta_x > 0$ and $\Delta_y < 0$ in this case). To relate these eigenvectors to the excitation voltage (amplitude and phase) for each loudspeaker, we need to multiply the eigenvectors by the coupling matrix $\mathbf{M}$ shown in Fig. S2 **d** and accounting for the frequency response of each speaker. The lower inset shows the amplitude (brightness) and phase (color) of the excitation voltage to be applied to each loudspeaker to generate the appropriate wavefront to optimally push the ball into the target position.

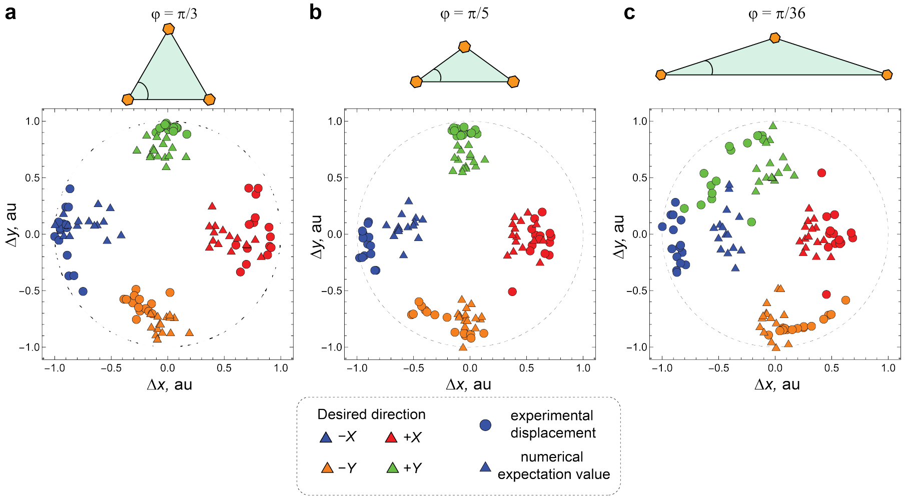

Fig. S6 Observed and calculated object's displacement for the Wigner-Smith operator for different zigzag triangles. We run a systematic experimental study where the three orange points are on an isosceles triangle with angle ϕ, and we ask for motion along $\pm X$ and $\pm Y$. **a**, When $\phi = \pi/3$, we compare the actual measured ball motion (disks) with what is expected from theory (i.e., in the absence of measurement noise): the accuracy of the method is very high, with the ball always moving in the right direction. This degrades at very small angles, such as **b**, $\phi = \pi/5$, or **c**, $\phi = \pi/36$. This study evidences the importance of accurately extracting the gradient of the $\boldsymbol{S}$ matrix by triangulating the motion about the desired path, as we do in this work.

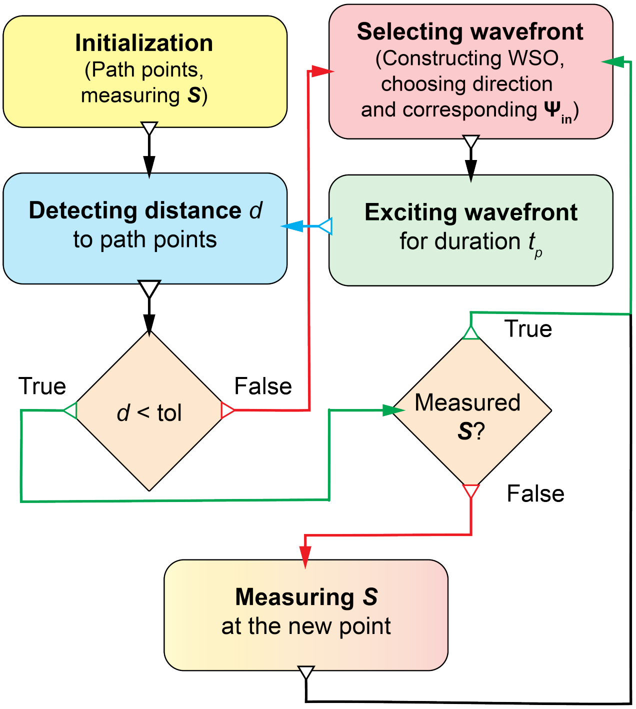

Fig. S7 Algorithm flow chart followed for the iterative object manipulation. We first discretize the target path into a zig-zag-like path and initialize the system by measuring the $\boldsymbol{S}$ matrices at the first three locations. From this, we start the algorithm, detect the ball's position, and estimate the distance to the nearest path point. If the distance from the path point is lower than a given tolerance value, the ball has attained a target position. We then check if we already measured $\boldsymbol{S}$ at this position. If this is the case (for example, just after initialization), we directly compute the GWS operator from the three previous scattering matrices. If we don't have a measure of $\boldsymbol{S}$ at the actual location, we first measure it and then compute the GWS operator. We select the optimal incident state by solving the eigenvalue problem and then excite the selected wavefront during a given time t_p by exciting the 20 speakers with the proper phase and amplitude voltages. Once the ball stabilizes, we start the algorithm and loop until the path is covered.

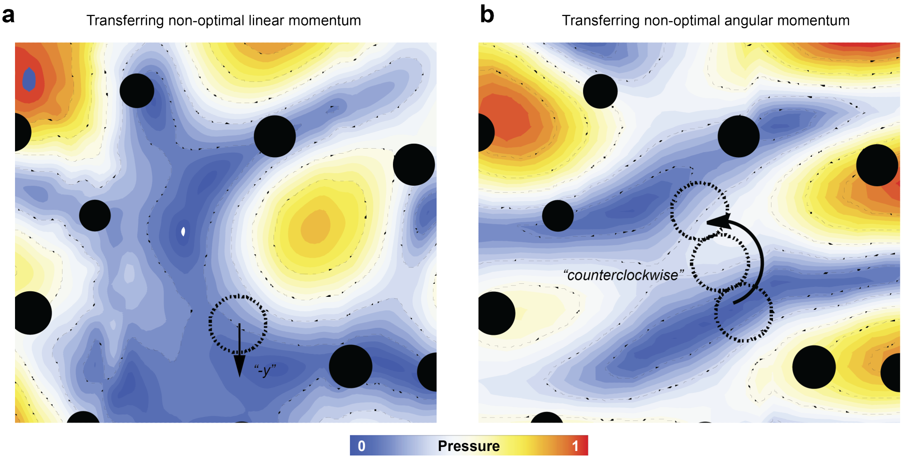

Fig. S8 Field map examples for non-optimal momentum transfer. We measured the field distribution excited by the wavefronts for eigenvectors corresponding to non-maximum eigenvalues in **a**, translation, and, **b**, angular momentum.

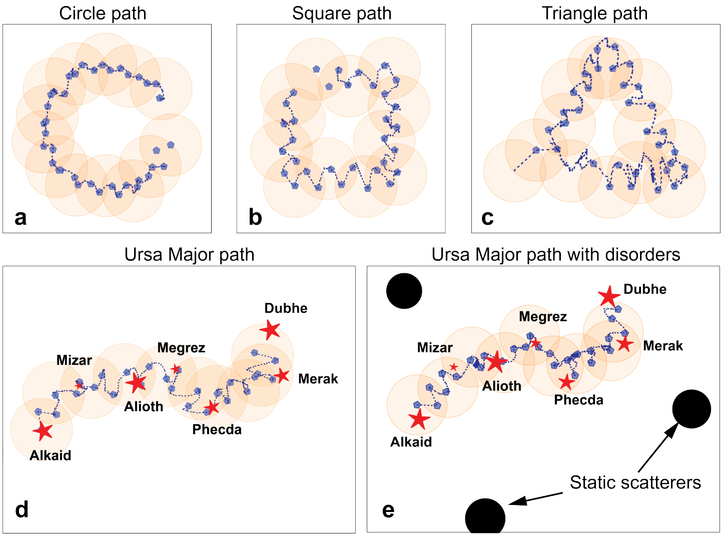

Fig. S9 Other trajectory examples. We first tested our method in the absence of scatterers on trajectories shaped like **a**, a circle, **b**, a square, **c**, a triangle, and **d**, the Ursa major star constellation. **d** We then repeated the case of the Ursa Major constellation in the presence of additional scatterers before trying the longer path shown in the main text.
